# Protective effect of tanshinone IIA against radiation-induced ototoxicity in HEI-OC1 cells

**DOI:** 10.3892/ol.2013.1486

**Published:** 2013-07-24

**Authors:** SHASHA DU, QIWEI YAO, PEIXIN TAN, GUOZHU XIE, CHEN REN, QUANQUAN SUN, XIAO ZHANG, RONG ZHENG, KAIJUN YANG, YAWEI YUAN, QUAN YUAN

**Affiliations:** 1 Department of Radiation Oncology, Institute of Neurosurgery, Nanfang Hospital, Southern Medical University, Guangzhou 510515, P.R. China; 2 Department of Neurosurgery, Institute of Neurosurgery, Nanfang Hospital, Southern Medical University, Guangzhou 510515, P.R. China; 3 Jules Stein Eye Institute, David Geffen School of Medicine, University of California, Los Angeles, CA 90095, USA

**Keywords:** radiation induced ototoxicity, House Ear Institute-Organ of Corti-1, tanshinone IIA

## Abstract

Radiotherapy is a highly efficient treatment method for nasopharyngeal carcinoma that is often accompanied by significant ototoxic side-effects. The inner ear hair cells are particularly prone to serious injury following radiotherapy. Tanshinone IIA is a transcription factor inhibitor that is extracted from the traditional herbal medicine, *Salvia miltiorrhiza* Bunge. The present study investigated the effects of tanshinone IIA treatment on radiation-induced toxicity in the HEI-OC1 hair cell line. Using an MTT assay and flow cytometry, the radiation-induced weakening of the cells was observed to be alleviated when the cells were pre-treated with tanshinone IIA. Radiation exposure promoted p65/nuclear factor (NF)-κB nuclear translocation and activated the p53/p21 pathway, two processes which play a significant role in radiation-induced cell apoptosis. However, pre-treatment of the cells with tanshinone IIA inhibited p65/NF-κB nuclear translocation and p53/p21 pathway activation. These results demonstrate that tanshinone IIA is capable of protecting cochlear cells from radiation-induced injury through the suppression of p65/NF-κB nuclear translocation and the p53/p21 signaling pathway.

## Introduction

Radiation therapy is the most effective treatment method for nasopharyngeal carcinoma. However, the auditory pathway inevitably suffers from radiation exposure during the therapy process. Therefore radiation-induced ear injury, particularly sensorineural hearing loss (SNHL), is a common complication following radiotherapy for patients with nasopharyngeal carcinoma (1–2). Thus far, no effective strategy has been proposed to treat radiation-induced SNHL, which seriously deteriorates the quality of life for these patients.

Generally, radiation-induced SNHL is regarded as a result of damage to the auditory sensory hair cells in the cochlea (3). Animal experiments using radiation regimens similar to those applied clinically have demonstrated cochlear hair cell degeneration in the absence of damage to vascular and supporting structures (4,5). Low *et al*(6) reported that the cochlear OC-K3 cell line demonstrated dose-dependent cell apoptosis and was identified to upregulate p53-related genes by micro-array studies under γ-radiation. The data suggest that radiation-induced cochlear hair cell death may play a role in SNHL.

Numerous studies have investigated the protective effects of agents against ototoxicity induced by antibiotics or cisplatin, but not radiation-induced ototoxicity. Currently there is no effective drug to protect against radiation-induced ototoxicity. Tanshinone is a derivative of phenanthrene-quinone with anti-oxidant and anti-inflammation properties that is isolated from *Salvia miltiorrhiza* Bunge (7–9). Tanshinone IIA significantly attenuates aminoglycoside-induced free radical formation *in vitro* and ototoxicity *in vivo*(10). Furthermore, tanshinone IIA is able to inhibit the radiation-induced activation of nuclear factor (NF)-κB in microglia BV-2 cells (11). Hence, the present study aimed to investigate the *in vitro* effect of tanshinone IIA on radiation-induced apoptosis and cell death in the cochlea. Using the HEI-OC1 cell line, the release of the apoptosis-inducing factors, p53 and p21, and the subsequent activation of the NF-κB pathway, were observed in the irradiated HEI-OC1 cells.

## Materials and methods

### Cell culture and radiation exposure

The HEI-OC1 cells were cultured in Dulbecco’s modified Eagle’s medium (DMEM), supplemented with 10% fetal bovine serum (FBS) at 33°C under 10% CO_2_ in an incubator. The HEI-OC1 cells were irradiated using a 6-MV linear accelerator (LINAC; 2300EX; Varian Co., Palo Alto, CA, USA) at a dose rate of 4.0 Gy/min. All the irradiations were performed at room temperature (18–25°C). Subsequent to being irradiated, the culture plates were returned to an incubator under the same conditions as previously described (33°C, 10% CO_2_).

### Cell viability assay

The cytotoxicity of tanshinone IIA was determined by an MTT assay. The HEI-OC1 cells were seeded in 96-well plates at a density of 1×10^4^ cells/well in 200 μl complete medium. Subsequent to being incubated overnight, the medium in each well was discarded and replaced with fresh medium containing various concentrations of tanshinone IIA (1–64 μg/ml). The cells that were not treated with tanshinone IIA were used as controls. Following a 24-h incubation period, 20 μl 5 mg/ml MTT was added to each well and cultivated for an additional 4 h. The supernatant was removed, 150 μl/well dimethyl sulfoxide (DMSO) was added and the samples were shaken for 15 min. The optical density (OD) was measured at 490 nm and the wells that did not contain cells were used as blanks. The protective effect of tanshinone IIA on radiation-induced ototoxicity was also determined using an MTT assay. The cells were incubated with or without tanshinone IIA and exposed to 0 and 16 Gy radiation, respectively. The cell viability was measured following irradiation for 24, 72 and 120 h. All the experiments were performed in triplicate.

### Cell morphology observation

The HEI-OC1 cells were irradiated and subsequently incubated for 72 h. Then, the cell morphology was observed by inversion microscopy. The nuclear morphology was observed under a fluorescence microscope (Olympus, Tokyo, Japan) subsequent to the cells being stained with 4′,6-diamidino-2-phenylindole (DAPI).

### Annexin V-fluorescin isothiocyanate/propidium iodide (FITC/PI) staining

Annexin V-FITC/PI staining was performed to quantify the percentage of apoptotic cells at 72 h post-irradiation. The cells were stained using the Annexin V-FITC Apoptosis Detection kit (Invitrogen, Inc., Carlsbad, CA, USA) following the manufacturer’s instructions. The cells were washed with phosphate-buffered saline (PBS) three times and resuspended in 1X binding buffer. Subsequently, the cells were incubated with Annexin V-FITC and PI for 10–15 min at room temperature and analyzed using flow cytometry.

### Colocalization of γH2AX and p65/NF-κB

The HEI-OC1 cells were planted onto polylysine-coated cover glasses and treated with irradiation. Subsequently, the samples were fixed in 4% paraformaldehyde for 30 min, permeabilized in 0.25% triton X-100 and blocked for 30 min in 1% goat serum. Subsequent to being blocked overnight at 4°C, anti-γH2AX primary antibody (1:100 dilution; Abcam Inc., Cambridge, MA, USA) and anti-p65/NF-κB primary antibody (1:200 dilution; CST Inc., Mount Carmel, IL, USA) were applied. Following this, the cells were washed three times for 5 min each in PBS, then rabbit anti-mouse AlexaFlour-488 secondary antibody (1:200 dilution) and goat anti-rabbit AlexaFluor-568 secondary antibody (1:200 dilution; Invitrogen, Inc.) were applied for 1 h at room temperature in the dark, followed by three 5-min washes in PBS. The samples were then mounted in fluorescence mounting medium with DAPI. The cells were observed using a fluorescence microscope (Olympus).

### RNA isolation and quantitative (q)PCR

Total RNA was purified from the cultured cells using TRIzol reagent (Invitrogen), according to the manufacturer’s instructions. Each RNA sample was reverse-transcribed to cDNA for 15 min at 37°C and 5 sec at 85°C using a PrimeScript^®^ RT reagent kit (Takara, Inc., Kyoto, Japan). qPCR was performed using the Stratgene MX3005P qPCR system with a SYBR^®^ Premix Ex Taq™ II kit (Takara Inc.). A total of 40 cycles of amplification were performed at 95°C for 30 sec, 60°C for 15 sec and 72°C for 15 sec. The fluorescence signal was detected at the end of each cycle. A melting curve analysis was used to confirm the specificity of the products. The 2^−ΔΔCt^ method was performed to analyze the results. The relative expression levels of each mRNA was assessed using the 2^−ΔΔCt^ method by normalizing to GAPDH and comparing it with the control samples.

### Western blot analysis

The cells were washed and suspended in lysis buffer (KeyGen Biotech, Inc., Nanjing, Jiangsu, China). The proteins were solubilized by sonication. Equal amounts of protein were separated by SDS-PAGE and transferred onto polyvinylidene difluoride membranes (Millipore Corp., Bedford, MA, USA). The membranes were blocked in PBS containing 0.1% Tween-20 and 5% powdered milk and probed with primary antibodies. Primary antibodies against phosphorylated-p21 (p-p21; Epitomics, Inc., Burlingame, CA, USA), p-p53 and β-actin (Bioworld technology, Inc., St Louis Park, MN, USA) were used at a dilution of 1:500.

### Statistical analysis

All values are presented as the mean ± SD. The data analysis was performed using the SPSS 13.0 statistical program (SPSS, Inc., Chicago, IL, USA). A one-way ANOVA was used to determine the statistical significance among the various groups and the LSD method was used for the pairwise comparisons. P<0.05 was considered to indicate a statistically significant difference.

## Results

### Effects of tanshinone IIA on the cell morphological changes and viability induced by irradiation

**Figure 1:**
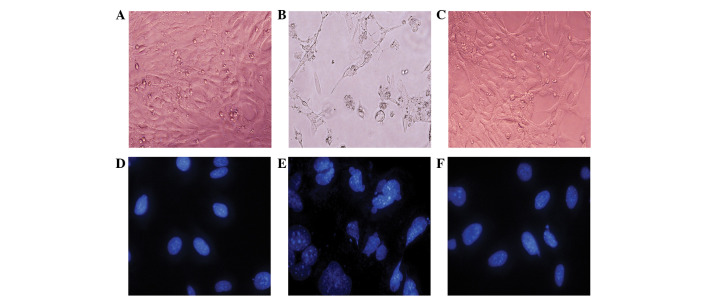
Morphological post-irradiation changes of the HEI-OC1 cells. (A) HEI-OC1 sham-irradiated cells showing a polygon and fusiform morphology. (B) The HEI-OC1 cells following 16-Gy irradiation for 72 h showing a dendritic or amoeboid appearance, with numerous highly ramified processes and body swelling. (C) The majority of the cochlear hair cells treated with tanshinone IIA and irradiation show a polygon and fusiform morphology, rather than a dendritic or amoeboid appearance. (D) The nuclei of the cells showing a uniform, oval shape following irradiation. (E) The cells were treated with 16 Gy. The cell nuclei exhibit shrinkage, fragmentation, megakaryocytes and deformation. (F) The cells were co-treated with tanshinone IIA and radiation and the nuclei appear to be oval-shaped and of a relatively homogeneous size. Magnification, (A–C) ×200; (D–F) ×400 (4′,6-diamidino-2-phenylindole staining).

In the absence of irradiation, the HEI-OC1 cells showed a normal polygon and fusiform morphology (Fig. 1A), with oval, uniformly sized nuclei (Fig. 1D). There was no significant morphological change in the HEI-OC1 cells following 2 or 4 Gy irradiation. However, when treated with 16 Gy, the HEI-OC1 cell morphology was changed to a dendritic or amoeboid appearance with numerous highly ramified processes and body swelling (Fig. 1B). The cell nucleus demonstrated shrinkage, fragmentation, megakaryocytes and deformation (Fig. 1E). In contrast, the majority of the irradiated HEI-OC1 cells that were pre-treated with tanshinone IIA preserved their polygon and fusiform morphology rather than change to the dendritic or amoeboid appearance (Fig. 1C), while the nuclei appeared to be oval-shaped and of a relatively homogeneous size (Fig. 1F).

**Figure 2:**
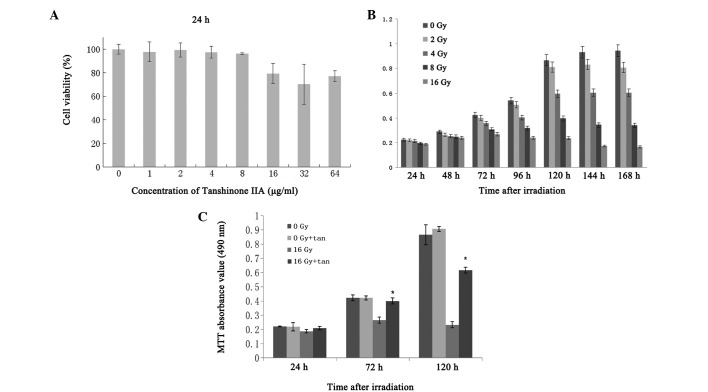
(A) Effect of tanshinone IIA on the cell viability of the HEI-OC1 cells. The HEI-OC1 cells were seeded into culture plates and treated with 1, 2, 4, 8, 16, 32 and 64 μg/ml tanshinone IIA for 24 h, then the cell viability was determined using an MTT assay. (B) The post-irradiation viability of the cells was dose dependent. MTT absorbance values for the different doses of irradiation at various time-points are shown. (C) Effect of tanshinone IIA on the cell viability of the HEI-OC1 cells. When the cultured cells were exposed to 16 Gy irradiation, the cell viability was 84.68, 62.88 and 26.91% following 24, 72 and 120 h of irradiation, respectively. However, when the cells were exposed to 16 Gy irradiation following treatment with 8 mg/ml tanshinone IIA, the cell viability was 94.59, 94.56 and 71.25% at the various time-points, respectively, and significantly higher than those without tanshinone IIA (P<0.01). There were no significant changes in the groups that were exposed to 0 Gy with or without tanshinone IIA at the various time-points. Each value represents the mean ± SD of three separate experiments.

To investigate the effect of tanshinone IIA on cell viability, the cells were exposed to tanshinone IIA for 24 h. As shown in Fig. 2A, the concentrations (1–8 μg/ml) of tanshinone IIA that were used had no effect on the viability of the HEI-OC1 cells. However, tanshinone IIA at concentrations of >16 μg/ml resulted in cytotoxicity. According to the preliminary results obtained from the MTT assay, the maximum concentrations of 8 μg/ml tanshinone IIA were not used for further experiments to avoid the tanshinone IIA cytotoxicity effect on the HEI-OC1 cell line. The results also showed that the irradiation decreased the viability of the HEI-OC1 cells in a time- and dose-dependent manner (Fig. 2B). To verify whether tanshinone IIA was able to prevent radiation-induced cytotoxicity, the HEI-OC1 cells were incubated with 8 μg/ml tanshinone IIA and exposed to 0 and 16 Gy irradiation. As shown in Fig. 2C, the treatment with tanshinone IIA significantly protected the HEI-OC1 cells from the irradiation.

### Tanshinone IIA inhibits radiation-induced apoptosis in HEI-OC1 cells

**Figure 3:**
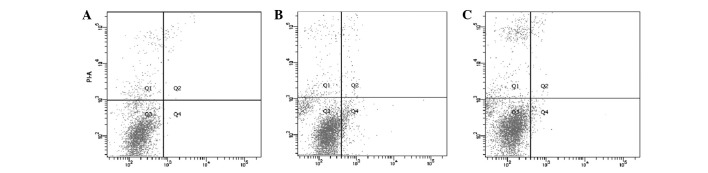
Flow cytometry to show the effect of irradiation and tanshinone IIA on cell apoptosis. (A) Irradiation of the HEI-OC1 cells resulted in few apoptotic cells. (B) Following 16-Gy irradiation for 72 h, the HEI-OC1 cells showed an increased apoptotic cell ratio. (C) The ratio of apoptotic cells was significantly reduced with irradiation with tanshinone IIA compared with irradiation alone. The results are representative of three separate experiments.

Flow cytometry was used to analyze the percentage of apoptotic cells that were treated with radiation in the absence or presence of tanshinone IIA (Fig. 3). Radiation accompanied by tanshinone IIA treatment significantly decreased the number of apoptotic cells (mean, 3.36%) compared with the cells that were treated with radiation only (mean, 9.09%; P<0.05), while there was no significant difference between the control group and the group with tanshinone IIA treatment alone. These results indicate that radiation promotes cell apoptosis and that radiation-induced apoptosis may be inhibited by a pre-treatment with tanshinone IIA.

### Effects of tanshinone IIA on radiation-induced p21 and p53 expression

**Figure 4:**
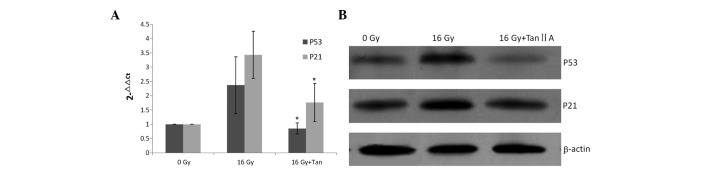
Effects of tanshinone IIA on the radiation-induced expression of p21 and p53. (A) The expression level of the p53/p21 genes was detected by qPCR. (B) p-p21/p-p53 was upregulated following irradiation, which was attenuated with tanshinone IIA treatment. The results are representative of three separate experiments. qPCR, quantitative PCR.

qPCR and western blotting were used to investigate the changes in p21 and p53 expression following irradiation with or without tanshinone IIA. As shown in Fig. 4, the levels of p21 and p53 after being stimulated by irradiation were significantly higher than those of the control group. When the cells were irradiated with tanshinone IIA, the upregulation of p21 and p53 was attenuated.

### Inhibitory effects of tanshinone IIA are mediated by p65/NF-κB pathway suppression in the radiation-induced DNA damage response

**Figure 5:**
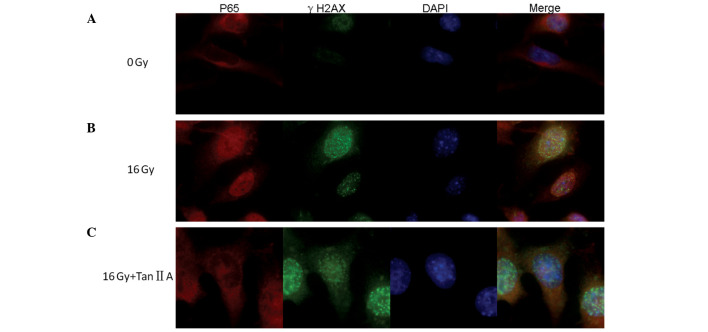
Post-irradiation expression of γ-H2AX and p65. Confocal images of immunofluorescence double staining for γ-H2AX and p65 in the HEI-OC1 cells following 16 Gy irradiation at 24 h with or without pretreatment with 8 mg/ml tanshinone IIA. DNA was counterstained with DAPI. Original magnification, ×400. (A) HEI-OC1 cell irradiation showing the minimal expression of γ-H2AX and the expression of p65 in the cytoplasm. (B) HEI-OC1 cells following 16 Gy irradiation showing the expression of γ-H2AX foci formation and showing that p65 is translocated to the nucleus. (C) HEI-OC1 cells following 16 Gy irradiation pre-treatment with tanshinone IIA showing the expression of γ-H2AX foci formation and showing that p65 is inhibited from translocating into the nucleus. DAPI, 4′,6-diamidino-2-phenylindole.

Double immunofluorescence staining for γH2AX and p65 was established to investigate the repair of double-strand breaks (DSBs) and the translocation of p65/NF-κB in the HEI-OC1 cells following irradiation (Fig. 5). The expression of the γH2AX foci was almost completely absent and that of the p65 was mainly located in the cytoplasm in the cells of the control group (Fig. 5A). Following 16 Gy irradiation for 6 h, distinct nuclear γH2AX foci were observed and the nuclei of the HEI-OC1 cells were stained red, indicating the translocation of p65 (Fig. 5B). However, when using the pre-treatment with 8 mg/ml tanshinone IIA, p65 protein translocation was inhibited and the cytoplasm was stained red (Fig. 5C)

## Discussion

Radiation damages tumor DNA by direct and/or indirect effects on the tumor cells (12), leading to cell apoptosis and cell death, which is the biological basis of radiotherapy. However, in the course of radiotherapy, normal tissues that surround the tumor are inevitably subjected to a certain dose of irradiation. Although a vast amount information is available with regard to the radiation-induced apoptosis and cell death of tumor cells, there is limited information on the effects of radiation on normal cells, particularly on the auditory hair cells of the cochlea. Furthermore, information with regard to the agents that may protect against radiation-induced ototoxicity is also limited.

In the present study, by observing the morphology of cells and using an MTT assay and flow cytometry, the cells with body swelling, nuclear fragmentation, decreased viability and an increased percentage of apoptotic cells were regarded as evidence of apoptosis and cell death in radiation-induced toxicity. This is consistent with the previous conclusion that ototoxicity induced by irradiation leads to cell apoptosis and death (3). In the present study, tanshinone IIA accompanied by irradiation treatment decreased the changes in radiation-induced cell morphology and cell viability. Furthermore, tanshinone IIA attenuated the irradiation-induced cell apoptosis.

Radiation induces the apoptotic death of various cells through the activation of a number of intracellular signaling pathways. NF-κB has been suggested to be activated in response to radiation-induced DNA damage. DSBs are the most deleterious form of DNA damage following ionizing radiation. H2AX phosphorylation is an early step in the response to DNA damage, and it has been verified that enumerating γH2AX foci may be a method used to measure the induction and repair of radiation-induced DSBs (13,14). Ataxia telangiectasia mutated (ATM) is a crucial component of the DNA DSB signaling cascade and has been suggested to be able to activate NF-κB in response to DNA damage (15,16). The NF-κB signaling pathway mediates a variety of significant cellular functions by regulating apoptosis and inflammatory responses. In unstimulated cells, NF-κB is in the form of a heterodimer of p65/p50 binding to the inhibitor proteins, namely Iκβ. Following stimulation, the release of p65/p50 from the Iκβ-α protein and the degradation of Iκβ-α are necessary for p65 translocation into the nucleus to regulate gene transcription. In the nucleus, NF-κB may induce a number of genes that activate intracellular programs, leading to either apoptosis or cell death. Among the apoptotic genes activated by NF-κB is p53, which may activate the transcription of other genes, including p21, to progress the apoptosis pathway (17). In the present study, it was identified that irradiation was able to activate the translocation of p65 and lead to an increase in the expression of p53/p21, which plays a significant role in apoptosis. The present study also indicated that tanshinone IIA exerted anti-apoptosis properties by suppressing the translocation of p65 and the transcription of p53/p21 through the NF-κB signaling pathway.

Although the results of the present study indicate that tanshinone IIA reduces radiation-induced ototoxicity, its clinical effect remains unclear. Since the present study was based on the HEI-OC1 cell line, which was harvested from the cochlea of immortal mice, tanshinone IIA is presumed to have an ability to protect the cochlea in radiation-induced ototoxicity. Future studies may focus on *in vivo* experiments to reveal the effect of tanshinone IIA on cochlea function.

To the best of our knowledge, this is the first study on auditory hair cells to investigate the protective effects of tanshinone IIA against radiation-induced ototoxicity. The results of the present study provide evidence of the anti-apoptotic effects of tanshinone IIA. Tanshinone IIA was also observed to enhance HEI-OC1 cell viability and prevent NF-κB translocation to inhibit p53/p21 activation when the cells underwent irradiation.

## References

[1] Chan SH, Ng WT, Kam KL (2009). Sensorineural hearing loss after treatment of nasopharyngeal carcinoma: a longitudinal analysis. Int J Radiat Oncol Biol Phys.

[2] Petsuksiri J, Sermsree A, Thephamongkhol K (2011). Sensorineural hearing loss after concurrent chemoradiotherapy in nasopharyngeal cancer patients. Radiat Oncol.

[3] Low WK, Burgess R, Fong KW, Wang DY (2005). Effect of radiotherapy on retro-cochlear auditory pathways. Laryngoscope.

[4] Kelemen G (1963). Radiation and ear. Experimental studies. Acta Otolaryngol.

[5] Gamble JE, Peterson EA, Chandler JR (1968). Radiation effects on the inner ear. Arch Otolaryngol.

[6] Low WK, Tan MG, Sun L (2006). Dose-dependent radiation-induced apoptosis in a cochlear cell-line. Apoptosis.

[7] Dong H, Mao S, Wei J (2012). Tanshinone IIA protects PC12 cells from β-amyloid(25–35)-induced apoptosis via PI3K/Akt signaling pathway. Mol Biol Rep.

[8] Zhu B, Zhai Q, Yu B (2010). Tanshinone IIA protects rat primary hepatocytes against carbon tetrachloride toxicity via inhibiting mitochondria permeability transition. Pharm Biol.

[9] Xia WJ, Yang M, Fok TF (2005). Partial neuroprotective effect of pretreatment with tanshinone IIA on neonatal hypoxia-ischemia brain damage. Pediatr Res.

[10] Wang AM, Sha SH, Lesniak W, Schacht J (2003). Tanshinone (Salviae miltiorrhizae extract) preparations attenuate aminoglycoside-induced free radical formation in vitro and ototoxicity in vivo. Antimicrob Agents Chemother.

[11] Dong X, Dong J, Zhang R (2009). Anti-inflammatory effects of tanshinone IIA on radiation-induced microglia BV-2 cells inflammatory response. Cancer Biother Radiopharm.

[12] Shinomiya N (2001). New concepts in radiation-induced apoptosis: ‘premitotic apoptosis’ and ‘postmitotic apoptosis’. J Cell Mol Med.

[13] Löbrich M, Rief N, Kühne M (2005). In vivo formation and repair of DNA double-strand breaks after computed tomography examinations. Proc Natl Acad Sci USA.

[14] Rübe CE, Dong X, Kühne M (2008). DNA double-strand break rejoining in complex normal tissues. Int J Radiat Oncol Biol Phys.

[15] Hadian K, Krappmann D (2011). Signals from the nucleus: activation of NF-kappaB by cytosolic ATM in the DNA damage response. Sci Signal.

[16] Wu ZH, Shi Y, Tibbetts RS, Miyamoto S (2006). Molecular linkage between the kinase ATM and NF-kappaB signaling in response to genotoxic stimuli. Science.

[17] Grilli M, Memo M (1999). Possible role of NF-kappaB and p53 in the glutamate-induced pro-apoptotic neuronal pathway. Cell Death Differ.

